# Potential impact on cholesterol goal achievement and predicted cardiovascular risk by the addition of ezetimibe and bempedoic acid on top of statins: a simulation from the SANTORINI study

**DOI:** 10.1016/j.ajpc.2026.101577

**Published:** 2026-03-24

**Authors:** Kausik K. Ray, Carlos Aguiar, Marcello Arca, Derek L. Connolly, Mats Eriksson, Jean Ferrières, Ulrich Laufs, Jose M. Mostaza, David Nanchen, Charles Boachie, Ben Lee, Jarkko Soronen, Christian Becker, Ernst Rietzschel, Timo Strandberg, Hermann Toplak, Frank L.J. Visseren, Alberico L. Catapano, on behalf of the SANTORINI study investigators

**Affiliations:** a Imperial Centre for Cardiovascular Disease Prevention, ICTU-Global, Imperial College London, London, UK; b Heart Institute, Carnaxide, Portugal; c Department of Translational and Precision Medicine, Sapienza Università di Roma, Rome, Italy; d The Midland Metropolitan University Hospital and the Birmingham City and Sandwell Health Campuses, Sandwell and West Birmingham NHS Trust, Birmingham, UK; e Karolinska University Hospital, Stockholm, Sweden; f Department of Cardiology and INSERM UMR 1295, Toulouse Rangueil University Hospital, Toulouse University School of Medicine, Toulouse, France; g University Hospital Leipzig, Leipzig, Germany; h La Paz-Carlos III Hospital, Madrid, Spain; i Center for Primary Care and Public Health (Unisanté), University of Lausanne, Lausanne, Switzerland; j Daiichi Sankyo Europe GmbH, Munich, Germany; k Ghent University and Ghent University Hospital, Ghent, Belgium; l University of Helsinki and Helsinki University Hospital, Helsinki, Finland, and University of Oulu, Center for Life Course Health Research, Oulu, Finland; m Department of Medicine, Division of Endocrinology and Diabetology, Medical University of Graz, Graz, Austria; n Department of Vascular Medicine, University Medical Center Utrecht, Utrecht, the Netherlands; o Department of Pharmacological and Biomolecular Sciences, University of Milan, Italy; p Multimedica IRCCS, Milan, Italy

**Keywords:** Bempedoic acid, Cardiovascular disease, Goal attainment, High cardiovascular risk, Low-density lipoprotein cholesterol, Lipid-lowering therapy, Simulation study

## Abstract

**Background:**

In SANTORINI, one-third of patients achieved low-density lipoprotein cholesterol (LDL-C) goals at 1-year follow-up, with oral combination therapies used in approximately 38 %. We simulated the potential impact of adding oral lipid-lowering therapies (LLTs) on goal attainment and predicted residual cardiovascular (CV) risk.

**Methods:**

Using observed LLT use and LDL-C levels as a baseline, a Monte Carlo simulation was applied. The predicted improvements in LDL-C through the sequential addition of ezetimibe (if not already received) and bempedoic acid (if not at goal with ezetimibe) was evaluated in patients not at goal (LDL-C simulation set; *N* = 6866), nor receiving background bempedoic acid or proprotein convertase subtilisin/kexin type 9 inhibitor therapies (treatment optimisation set; *N* = 4467). Predicted 10-year CV risk reduction from expected LDL-C changes were also assessed (CV risk simulation set; *N* = 4327).

**Results:**

Adding ezetimibe and ezetimibe plus bempedoic acid increased the predicted number of patients at goal from 28.4 % to 43.8 % and 63.1 %, respectively. The predicted relative risk reductions of a major CV event was 7.6 % and 13.4 % after each treatment optimisation step. Relative to the predicted residual 10-year risk of 25.1 % at the end of SANTORINI, further LLT optimisation was predicted to lower absolute CV risk by 2.0 % and 3.4 % with each additional LLT optimisation step.

**Conclusions:**

Approximately two-thirds of patients could reach their LDL-C targets after the stepwise addition of ezetimibe and bempedoic acid in patients not at goal with statins and/or ezetimibe, with likely meaningful CV risk reductions over 10 years.

**Funding:**

This study was funded by Daiichi Sankyo Europe GmbH, Munich, Germany.

**Trial registration:**

ClinicalTrials.gov identifier: NCT04271280

## 1 Introduction

Cardiovascular (CV) disease is the leading cause of death, accounting for 17.9 million deaths globally and 3.9 million deaths in Europe each year [1,2]. It is well established that elevated low-density lipoprotein cholesterol (LDL-C) is causally linked to atherosclerotic cardiovascular disease (ASCVD) [[3], [4], [5], [6], [7], [8]].

Despite the prevalence of established lipid-lowering therapies (LLTs) in Europe, observational studies have shown that patients at high or very high CV risk fail to achieve their risk-based LDL-C goals [[9], [10], [11], [12], [13]]. Moreover, many patients receive suboptimal treatment in routine clinical practice and remain at an increased risk of CV events. This highlights the need for LLT intensification with combination therapies to reach a patient’s risk-based LDL-C goal [13,14].

Combination therapies that are based on the association of statins with ezetimibe result in better goal attainment and better CV outcomes in high- or very high-risk patients [[15], [16], [17]]. The addition of proprotein convertase subtilisin/kexin type 9 inhibitors (PCSK9is) may provide further benefit as they lead to risk-based LDL-C goal achievement in 90 % of patients as well as improved CV outcomes [[18], [19], [20]]. When considering treatment options, oral therapy is often preferred over injectable therapy owing to lower acquisition costs and ease of use, without training needed for self-injection (monoclonal antibodies) or administration by healthcare providers in a clinical setting (inclisiran). Hence, injectable treatments are restricted to patients who derive the largest absolute benefits to justify costs [21].

The Treatment of High and Very high riSk dyslipidemic pAtients for the preveNTion of cardiOvasculaR Events in Europe – a multInatioNal observatIonal study (SANTORINI) 1-year follow-up study reported any LLT usage of 79.2 % at baseline, which increased to 95.0 % at the 1-year follow-up, and combination therapy with statin and ezetimibe increased from 25.6 % to 37.9 % at the 1-year follow-up. Consequently, LDL-C goal attainment increased from 21.2 % at baseline to 30.9 % at the 1-year follow-up. While overall LLT usage improved in this population, risk-based LDL-C goal attainment only increased by approximately 10 % [22].

The objectives of this simulation study, which utilised the 1-year follow-up cohort of patients from the European SANTORINI study [22], were to evaluate the extent to which add-on oral therapies might improve the achievement of guideline-recommended LDL-C goals. In addition, the potential residual risk, absolute risk reduction (ARR) and relative risk reduction (RRR) of CV events predicted after treatment optimisation were also simulated.

## 2 Materials and methods

### 2.1 SANTORINI cohort at 1-year follow-up

The rationale and methods used in SANTORINI have been described previously [23]. SANTORINI was a multinational, multicentre, non-interventional study (NCT04271280) [13,23].

**Fig. 1:**
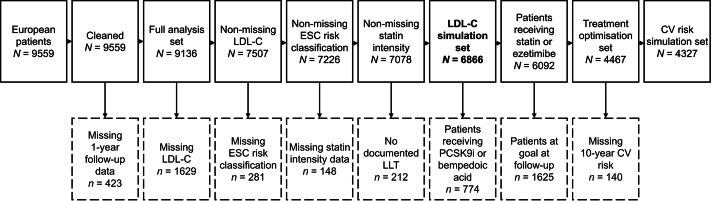
Selection of eligible patients from the European SANTORINI cohort at 1-year follow-up.

Abbreviations: CV, cardiovascular; ESC, European Society of Cardiology; LDL-C, low-density lipoprotein cholesterol; LLT, lipid-lowering therapy; PCSK9i, proprotein convertase subtilisin/kexin type 9 inhibitor.

Patients at high or very high CV risk (according to the physician) were recruited from 14 countries: Austria, Belgium, Denmark, Finland, France, Germany, Ireland, Italy, Portugal, Spain, Sweden, Switzerland, the Netherlands and United Kingdom [13]. Patients were eligible for inclusion if they received any LLT, had a known statin regimen (if receiving statins), and had a known LDL-C value (directly recorded or recalculated using the Friedewald formula; Fig. 1) at the 1-year follow-up visit. For the simulation, patients were stratified into high and very high CV risk groups using the risk classifications computed according to the 2019 European Society of Cardiology/European Atherosclerosis Society (ESC/EAS) guidelines [24].

Patient characteristics and LLT use at the 1-year follow-up were based on data available at the cut-off date of 31 May 2022. Data on routine management since baseline were also documented. Continuous variables were presented as mean (standard deviation [SD]), and categorical variables were presented as number of patients (percentage).

### 2.2 Simulation of the LLT pathway and LDL-C reduction

The LDL-C simulation set (*N* = 6866) was a subset of patients in the European SANTORINI cohort receiving LLT at the 1-year follow-up with available LDL-C measurement, CV risk classification, statin intensity and goal attainment data. A Monte Carlo simulation approach was used to simulate, as described previously, the sequential addition of ezetimibe (if not already received) and bempedoic acid (if not at goal after the addition of ezetimibe) [[25], [26], [27]]. Use of the 1-year follow-up data (instead of the baseline data) was selected to allow more patients to achieve stable LLT treatment before the data were input into the simulation [22].

The CV risk category and LDL-C treatment targets were based on the 2019 ESC/EAS guidelines on dyslipidaemia (<70 mg/dL for high-risk and <55 mg/dL for very high-risk patients), the 50 % relative decrease in LDL-C criterion was not considered [24].

CV risk was recalculated based on individual patient characteristics and application of the CV risk classification from the 2019 ESC/EAS dyslipidaemia guidelines for patients at high and very high risk. To allow simulation of LDL-C reduction and goal attainment, patients were excluded if CV risk classification could not be calculated, if they had unknown statin intensity or had no LDL-C measurements.

The simulated treatment algorithm was then applied (as described above) in patients who were not at goal with statins and/or ezetimibe and not receiving background bempedoic acid nor PCSK9i at the 1-year follow-up (treatment optimisation set; *N* = 4467). No statin intensification was simulated as it was assumed that at the 1-year follow-up, patients on LLTs were stable at their maximally tolerated regimen [27].

The efficacy of ezetimibe was simulated in a similar way to that reported by Cannon et al. [25] using a beta distribution with a mean LDL-C reduction from a baseline of 22.7 % [17] and SD of 16.5 % [28]. However, the alpha and beta parameters of the distribution used were not reported by Cannon et al. [25]. In this simulation, a beta distribution with alpha of 1.6 and beta of 5.4 was used, which provided a reasonable approximation of the reported treatment effect (mean, 22.9 %; SD, 14.8 %).

The efficacy of bempedoic acid was simulated, as previously reported, using log-normal distributions derived from the treatment effects observed in the Cholesterol Lowering via Bempedoic Acid, an ACL-Inhibiting Regimen (CLEAR) programme, for which patient data are accessible. Data were split into two pools: Pool 1 (CLEAR Harmony and Wisdom studies)—where the majority of patients were treated with maximally tolerated moderate- or high-dose statin therapy (mean LDL-C reduction, 16.7 %; SD, 20.9 %) [29,30] and Pool 2 (CLEAR Serenity and Tranquility studies)—patients treated with low-dose or no statin therapy (mean LDL-C reduction, 24.1 %; SD, 22.3 %) [31,32].

In the Monte Carlo simulation, depending on their background statin therapy at the 1-year follow-up, the corresponding SANTORINI patients used either Pool 1 (if on moderate- or high-dose statins) or Pool 2 (if on low-dose or no statin therapy) to simulate the reduction in LDL-C with bempedoic acid.

**Fig. 2:**
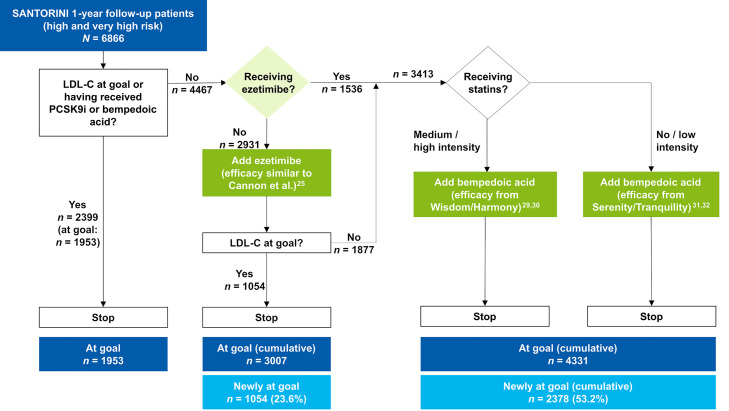
Simulation algorithm in the LLT pathway.

Abbreviations: LDL-C, low-density lipoprotein cholesterol; LLT, lipid-lowering therapy; PCSK9i, proprotein convertase subtilisin/kexin type 9 inhibitor.

The treatment effect on the LDL-C levels was simulated through a Monte Carlo simulation and run 10,000 times. All simulations were performed using R version 4.0.3 (2020) and run according to the LLT intensification algorithm (Fig. 2). The mean LDL-C value was calculated after the addition of ezetimibe and after the addition of bempedoic acid per 10,000 simulations. The median, 2.5 % quantile and 97.5 % quantile of the 10,000 LDL-C means and the number of patients at goal were estimated.

### 2.3 Calculation of residual CV risk

Residual CV risk at the 1-year follow-up, ARR and RRR after treatment optimisation was calculated for patients at high or very high CV risk who were not at their ESC/EAS guideline-recommended LDL-C goal at the 1-year follow-up and who were without missing data on the parameters needed to calculate the observed 10-year CV risk.

The predicted 10-year risk at the 1-year follow-up before treatment optimisation was calculated using the observed patient characteristics. In Supplementary Fig. 1, our approach is described by a CONSORT diagram. For patients with ASCVD, the REduction of Atherothrombosis for Continued Health (REACH) equation was used to estimate the 10-year risk of a CV event [33]. For patients without ASCVD, the Systematic COronary Risk Evaluation (SCORE) 2 and SCORE2-Older Persons (SCORE2-OP) approaches were used to estimate the 10-year risk of a CV event. SCORE2 and SCORE2-OP were retrospectively calculated for all patients based on the underlying model [34,35]. The SCORE2 diabetes model was not used because it included additional covariates, which were often missing in SANTORINI patients. Notably, although the SANTORINI study protocol considered the SCORE approach to calculate the 10-year risk of a CV event, CV risk was recalculated in this simulation study using SCORE2 to better reflect current medical practice [33,34]. The predicted 10-year CV risk, calculated with REACH and SCORE, is shown in Supplementary Table 1. Information on the performance of the REACH score in the SANTORINI dataset are provided in Supplementary Table 2 and Supplementary Fig. 2. The 20-month risk of a subsequent CV event (as predicted by the REACH equation) was converted into a 10-year risk; a constant rate over time (exponential survival function) was assumed [33].

The reduction in the 10-year predicted risk after treatment optimisation was estimated via a Monte Carlo process using the simulated LDL-C reduction, and random sampling from the normal distribution was associated with a rate ratio of 0.78 per 1.0 mmol/L (38.7 mg/dL) from the Cholesterol Treatment Trialists’ Collaboration (CTTC) meta-analysis [3]. The ARR and RRR in LDL-C, achieved by treatment optimisation for each patient, were also calculated.

As this was a retrospective analysis of de‑identified patient data, neither informed consent nor ethics approval was required.

## 3 Results

### 3.1 Study populations

Starting with data from the full analysis set of the European SANTORINI cohort at the 1-year follow-up (*N* = 9136) [22], 1629 patients who did not have an LDL-C value at the 1-year follow-up were excluded (Fig. 1). Two hundred and eighty-one patients were excluded for whom ESC risk classification was missing, and 148 patients were excluded for whom statin intensity data was missing. A further 212 patients who did not receive LLT at the 1-year follow-up were excluded. Hence, 6866 patients who met the eligibility criteria were selected and included in the LDL-C simulation set (Fig. 1). The effect of treatment optimisation on LDL-C was then simulated in patients who were not at goal and not receiving bempedoic acid or PCSK9i at the 1-year follow-up (*N* = 4467; treatment optimisation set). Finally, the effect of treatment optimisation on CV risk was simulated (*N* = 4327; CV risk simulation set) in patients where REACH, SCORE2 or SCORE2-OP could be calculated based on the patient characteristics (Table 1).

**Table 1:** Patient characteristics of the European SANTORINI cohort (*N* = 9136) and LDL-C simulation set (*N* = 6866) at 1-year follow-up.

Characteristics	SANTORINI cohort (*N* = 9136)	LDL-C simulation set (*N* = 6866)
Age, years, mean (SD)	66.4 (10.9)	66.4 (10.7)
Female, *n* ( %)	2489 (27.2)	1812 (26.4)
Diabetes mellitus, *n* ( %)	3316 (36.3)	2610 (38.0)
BMI, kg/m^2^, mean (SD)	28.2 (4.9)	28.2 (4.8)
LDL-C, mg/dL, mean (SD)	76.9 (36.5)	74.3 (33.9)
High CV risk, *n* ( %)^a^	2496 (27.3)	1690 (24.6)
Very high CV risk, *n* ( %)^a^	6307 (69.0)	5097 (74.2)
Primary prevention, *n* ( %)	2236 (24.5)	1499 (21.8)
Secondary prevention, *n* ( %)	6900 (75.5)	5367 (78.2)
MI, *n* ( %)	3882 (42.5)	3079 (44.8)
Unstable angina, *n* ( %)	1081 (11.8)	846 (12.3)
Stroke, *n* ( %)	611 (6.7)	465 (6.8)
TIA, *n* ( %)	393 (4.3)	307 (4.5)
FH, *n* ( %)	946 (10.4)	784 (11.4)

a Risk categories were assigned by the investigator. Abbreviations: BMI, body mass index; CV, cardiovascular; LDL-C, low-density lipoprotein cholesterol; FH, familial hypercholesterolaemia; MI, myocardial infarction; SD, standard deviation; TIA, transient ischaemic attack.

Patient use of LLTs in the SANTORINI and LDL-C simulation cohorts is presented in Table 2.

**Table 2:** Lipid-lowering therapy for the European SANTORINI cohort (*N* = 9136) and LDL-C simulation set (*N* = 6866) at 1-year follow-up.

Treatment	SANTORINI cohort (*N* = 9136)	LDL-C simulation set (*N* = 6866)
No statin users at follow-up	1973 (21.6)	641 (9.3)
Statins (any)	7163 (78.4)	6225 (90.7)
Low intensity	166 (1.8)	135 (2.2)
Moderate/high intensity	6930 (75.9)	6090 (97.8)
Statins alone	4812 (52.7)	3592 (52.3)
Ezetimibe alone	146 (1.6)	118 (1.7)
Bempedoic acid	64 (0.7)	55 (0.8)
PCSK9i alone	202 (2.2)	183 (2.7)
PCSK9i combination	600 (6.6)	536 (7.8)

Data are presented as *n* ( %).

Abbreviations: LDL-C, low-density lipoprotein cholesterol; PCSK9i, proprotein convertase subtilisin/kexin type 9 inhibitor.

### 3.2 Predicted change in LDL-C levels

In the LDL-C simulation set, the patient characteristics were similar to those of the European SANTORINI cohort at the 1-year follow-up (Table 1). Mean (SD) age was 66.4 (10.7) years; 26.4 % of the patients were female and mean (SD) LDL-C was 74.3 (33.9) mg/dL (Table 1). Approximately half of the patients (52.3 %; *n* = 3592/6866) were taking statin monotherapy; 1.7 % (*n* = 118/6866) ezetimibe monotherapy, and 0.8 % (*n* = 55/6866) bempedoic acid (Table 2). CV risk was calculated based on the individual patient characteristics from the 2019 ESC/EAS guidelines; it was high in 7.5 % of patients and very high in 92.5 % of patients.

**Fig. 3:**
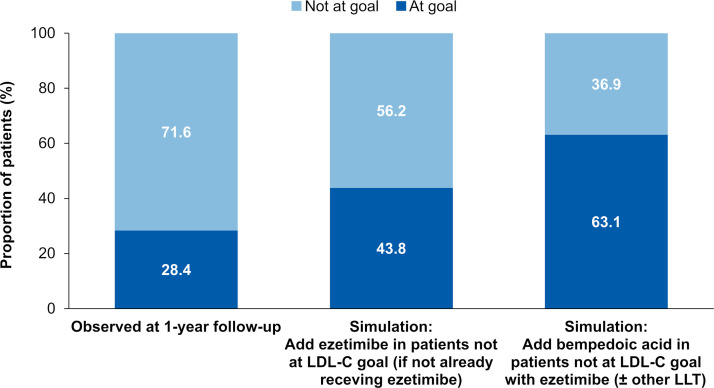
Cumulative LDL-C goal attainment before and after sequential simulation of ezetimibe and bempedoic acid plus ezetimibe (LDL-C simulation set; *N* = 6866).

Abbreviations: LDL-C, low-density lipoprotein cholesterol; LLT, lipid-lowering therapy.

Before simulation, 24.8 % and 28.7 % of high- and very high-risk patients, respectively, in the LDL-C simulation set were at their individual 2019 ESC-recommended LDL-C goals (Supplementary Fig. 3). Overall, LDL-C goal attainment in the LDL-C simulation set was 28.4 % (*n* = 1953/6866), which increased to 43.8 % (*n* = 3007/6866 [95 % confidence interval (CI): 43.2–44.4]) after simulating the additional LDL-C reduction attainable with ezetimibe in patients who were not at goal with statins alone (Figs. 2 and 3); mean LDL-C was 65.8 mg/dL with a mean predicted absolute change in LDL-C of −8.5 mg/dL (95 % CI: −8.7 to −8.3). After simulating the impact of added bempedoic acid among patients who were not at goal after ezetimibe alone, or statins and ezetimibe, overall goal attainment was predicted to increase to 63.1 % (*n* = 4331/6866 [95 % CI: 62.3–63.9]) (Figs. 2 and 3); mean LDL-C was 58.7 mg/dL with a mean predicted absolute change in LDL-C of −15.6 mg/dL (95 % CI: −15.2 to −15.9). The additional percentage of patients expected to reach goal after the sequential addition of ezetimibe and bempedoic acid was 34.7 % (Figs. 2 and 3).

**Fig. 4:**
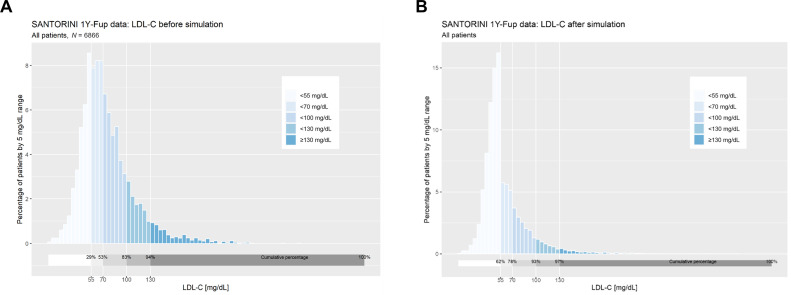
(A) LDL-C values before simulation. (B) LDL-C values after simulation.

Abbreviations: 1Y-Fup, 1-year follow-up; LDL-C, low-density lipoprotein cholesterol.

Patient distribution of LDL-C levels at 1-year follow-up before and after simulation are presented in Figs. 4a and 4b, respectively. Before simulation, 53 % of patients achieved an LDL-C concentration of <70 mg/dL, and 29 % achieved an LDL-C concentration of <55 mg/dL (Fig. 4a). At the end of the simulation, 78 % of patients were predicted to achieve an LDL-C concentration of <70 mg/dL and 62 % of patients were predicted to achieve an LDL-C concentration of <55 mg/dL (Fig. 4b).

### 3.3 Added benefit of each additional LLT regimen

Patient characteristics and LLT use for the treatment optimisation set are described in Supplementary Table 3 and Supplementary Table 4. The study design is presented in Supplementary Fig. 4. Stepwise intensification was simulated only in patients receiving statin and/or ezetimibe (i.e., excluding those already receiving PCSK9i or bempedoic acid) at the 1-year follow-up.

The addition of ezetimibe among patients not at goal at the 1-year follow-up improved goal attainment from 0 % to 23.6 % (*n* = 1054/4467 [95 % CI: 22.7–24.5]). Adding bempedoic acid (if still not at goal after ezetimibe) resulted in an additional 1324 patients newly at goal, thus increasing goal attainment from 0 % to 53.2 % (*n* = 2378/4467 [95 % CI: 52.1–54.4]) in the treatment optimisation set.

### 3.4 Predicted improvements in CV risk

**Fig. 5:**
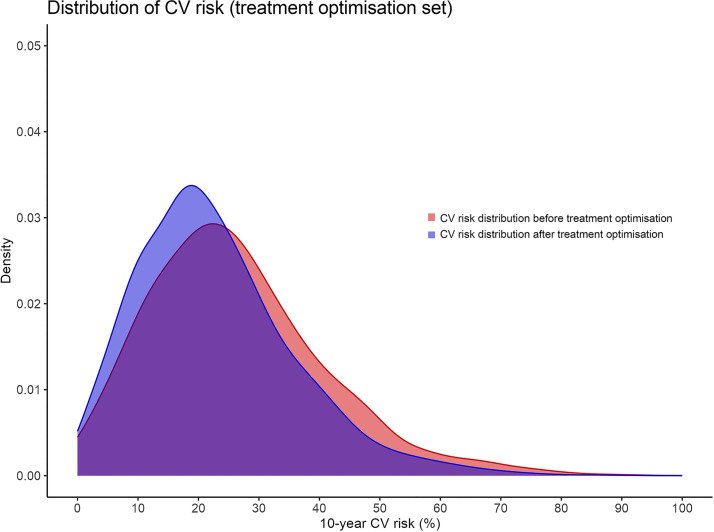
Distribution of CV risk (treatment optimisation set; *N* = 4467): derived using REACH, SCORE2 and SCORE2-OP.

Abbreviations: CV, cardiovascular; REACH, REduction of Atherothrombosis for Continued Health; SCORE, Systematic COronary Risk Evaluation; SCORE2-OP, SCORE2-Older Persons.

In the CV risk simulation set (*N* = 4327) (i.e., those not at goal and for whom 10-year CV risk could be calculated using observed patient characteristics), before simulation, 35 % of patients achieved an LDL-C concentration of <70 mg/dL, and 2 % achieved an LDL-C concentration of <55 mg/dL (Supplementary Fig. 5). The median predicted 10-year risk before treatment optimisation was 25.1 % (with 95 % quantiles of 3.1 % and 62.3 %; Fig. 5). The predicted mean RRR of major CV events was 7.6 % (95 % CI: 7.0–8.1) after treatment optimisation with ezetimibe and 13.4 % (95 % CI: 12.4–14.3) after the addition of bempedoic acid for patients who were predicted to remain above their LDL-C goal despite ezetimibe treatment (Supplementary Table 1).

The mean ARR predicted with this approach for a major CV event was 2.0 % (95 % CI: 1.8–2.1; corresponding to a number needed to treat [NNT] of 50) after treatment optimisation with ezetimibe, and 3.4 % (95 % CI: 3.2–3.7; NNT: 29.4) after treatment optimisation with ezetimibe and bempedoic acid (Supplementary Table 1).

Stratification by ESC/EAS risk categories (high risk: *n* = 311 [7.2 %]; very high risk: *n* = 4016 [92.8 %]) indicated that, following further treatment optimisation with ezetimibe alone, the predicted mean 10-year ARR for a major CV event was 1.0 % (95 % CI: 0.09–1.2; NNT: 100) for high-risk patients and 2.0 % (95 % CI: 1.9–2.2; NNT: 50) for very high-risk patients. After treatment optimisation with ezetimibe and bempedoic acid, the predicted mean 10-year ARR for a major CV event was estimated to be 1.6 % (95 % CI: 1.4–1.8; NNT: 62.5) in high-risk patients and 3.6 % (95 % CI: 3.3–3.8; NNT: 27.8) in very high-risk patients.

## 4 Discussion

The results from this simulation study suggest that the gap between LDL-C guideline recommendations and their achievement could be significantly improved through implementing treatment optimisation strategies with adjunctive use of currently available oral LLTs. In this regard, we found that adding oral combination therapy with ezetimibe and bempedoic acid to statins doubled the number of patients who achieved their risk-based LDL-C goals (from 28.4 % before simulation to 63.1 % at the end of the simulation; Central illustration). Potential reasons for the lack of goal attainment in routine clinical practice may include lowering of the ESC/EAS LDL-C goals in 2019, which places goal attainment out of reach for most patients taking statin monotherapy, even at the highest dose. Furthermore, factors such as intolerance to more effective doses of statins, a lack of perception of the additional benefits offered by further LDL-C lowering, the cost and availability of therapies such as ezetimibe, and limited access to PCSK9is could also affect population-level control [14,18]. Moreover, at the time of the SANTORINI study, bempedoic acid was the newest oral treatment option and was not widely available.

**Central illustration:**
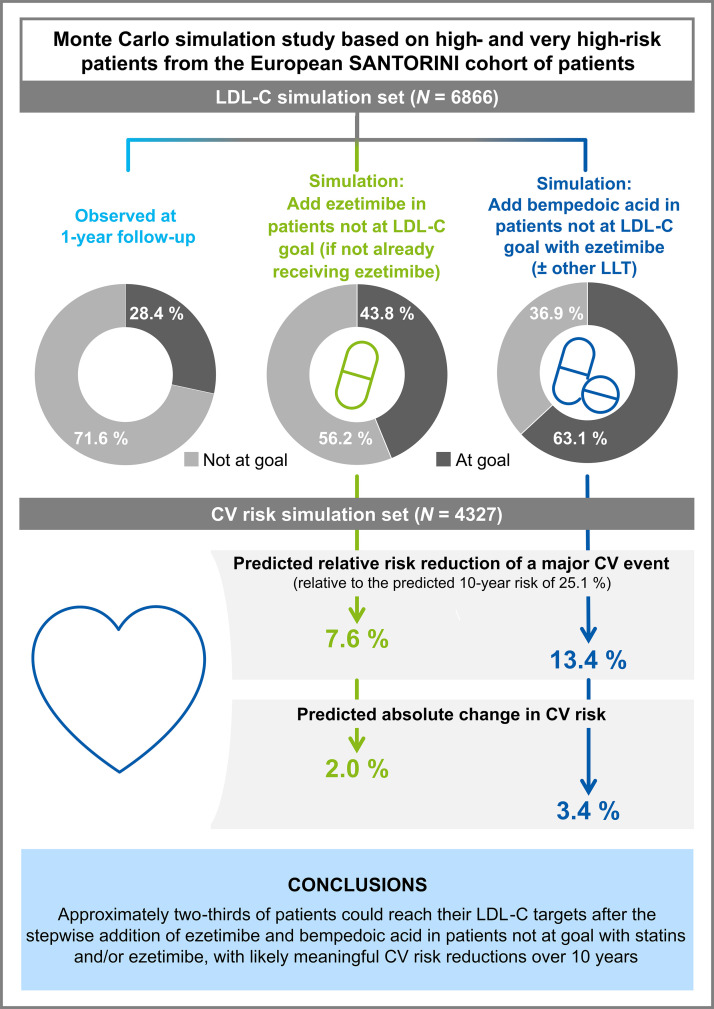
Effect of sequential addition of oral ezetimibe and bempedoic acid on LDL-C goal attainment and predicted CV risk. Abbreviations: CV, cardiovascular; LDL-C, low-density lipoprotein cholesterol; LLT, lipid-lowering therapy.

The results in the LDL-C simulation set indicate that further optimising oral add-on LLT in the SANTORINI population at the 1-year follow-up was associated with a predicted decrease in LDL-C from 74.3 mg/dL before simulation to 58.7 mg/dL at the end of the simulation, with 78 % and 62 % of patients achieving an LDL-C concentration of <70 mg/dL and <55 mg/dL, respectively. The benefits of treatment optimisation become increasingly evident when focusing on the population in the treatment optimisation set, in which goal attainment increased from 0 % to 53.1 % in patients who were not at their LDL-C goal at the 1-year follow-up. These simulation results are supported by real-world data, that show substantial increases in goal attainment following the initiation of bempedoic acid or bempedoic acid plus ezetimibe fixed-dose combination [36]. Moreover, from the proportion of patients who remain above goal after the final simulation step, it is possible to infer the number of patients who are likely to achieve goals with injectable therapies, which is approximately one-third of the SANTORINI cohort [37].

The RRR associated with further LDL-C lowering is assumed to be proportional to the absolute magnitude of LDL-C reduction, in line with the CTTC framework [3,38]. Consequently, patients with higher baseline LDL-C levels may experience greater RRRs because the LDL-C reduction in terms of absolute change would be expected to be larger in this group. A greater RRR would also be expected to provide greater ARR. However, patients with lower baseline LDL-C but higher overall CV risk due to comorbidities such as diabetes, chronic kidney disease or established ASCVD may have comparable ARRs and NNTs despite a smaller relative reduction in LDL-C [[39], [40], [41]]. This is consistent with findings from McQueen et al., who modelled the CV benefit of adding bempedoic acid plus ezetimibe fixed-dose combination versus ezetimibe alone in patients with ASCVD not at goal despite maximally tolerated statin therapy across a range of baseline LDL-C levels. The predicted absolute reduction in CV events increased from 4.9 % in patients with baseline LDL-C of 101–110 mg/dL to 10.9 % in those with baseline LDL-C exceeding 200 mg/dL [42].

Although a greater proportion of patients may achieve their cholesterol goals with additional oral medications, based upon this simulation data, approximately one-third of patients would not achieve their individual LDL-C goals after the addition of ezetimibe and bempedoic acid, which suggests that these patients would probably benefit from further LLT, such as the addition of PCSK9is [43]. However, optimising the use of ezetimibe and bempedoic acid may be a pragmatic first step in identifying patients with persistent unmet needs, before considering escalation to more resource-intensive and expensive LLTs, such as PCSK9is. Nonetheless, treatment decisions should remain individualised and aligned with the 2025 Focused Update of the 2019 ESC/EAS dyslipidaemia guidelines [37].

It was shown in a CTTC meta-analysis that a decrease in LDL-C by 1.0 mmol/L (38.7 mg/dL) reduced the risk of major CV events by approximately 22 % [3]. The CV risk reduction observed in the Improved Reduction of Outcomes: Vytorin Efficacy International (IMPROVE-IT) trial and the CLEAR Outcomes trial was generally consistent with the results of the CTTC meta-analysis [3,17,[44], [45], [46]]. Based on the CLEAR Outcomes trial, CV risk reduction with bempedoic acid in terms of the CTT endpoint of a ‘major vascular event’ was similar to that achieved with statins for a given absolute magnitude of LDL-C lowering [46]. Moreover, when the impact of LDL-C was assessed in patients with less atherosclerosis and lower event rates, the relative benefits per unit change in LDL-C were greater, suggesting that early intervention in the disease process provides a greater return from any given change in LDL-C [[46], [47], [48], [49], [50]].

Based on the findings from the CTTC meta-analysis [3], this simulation study indicates that optimised oral LLT with ezetimibe and bempedoic acid could substantially reduce CV risk (i.e., by approximately 34 major CV events in 1000 patients over 10 years). These results emphasise the potential value of combination therapy in lowering LDL-C beyond that achievable with statins alone and in potentially further reducing CV events in patients at high or very high CV risk. That said, even with additional LDL-C lowering, patients remain at a high risk of predicted CV events with optimised LLT, which suggests the need to consider additional treatment with PCSK9i, and risk factor modification much earlier, as recommended by the World Heart Foundation cholesterol roadmap [15,51].

The 2019 ESC/EAS guidelines recommend a stepwise approach to reach lipid goals [24], but this delays treatment for patients at high or very high CV risk who could benefit. The SWEDEHEART registry showed that reducing LDL-C by >1.0 mmol/L (38.7 mg/dL) in the first 3 months after an acute coronary syndrome (ACS) event led to a better prognosis than lowering LDL-C by 0.5 mmol/L (19.3 mg/dL) [52]. Moreover, it was recently suggested that intensive and sustained non-high-density lipoprotein cholesterol lowering may be more beneficial than a stepwise approach, where target achievement would be expected to occur later [53].

Early and intensive LDL-C reduction should be considered in specific contexts, such as ACS. Patients presenting with ACS are a particularly high-risk group in whom rapid and substantial LDL-C lowering is associated with improved CV outcomes [37]. Data from the PL-ACS registry, a large national prospective observational study of over 38,000 patients with ACS, demonstrated that upfront combination therapy with a statin and ezetimibe was superior to statin monotherapy for lowering all-cause mortality, with an ARR of 4.7 % and an NNT of 21 over 3 years [54]. This was further shown in the LAI-REACT study, in which triple combination therapy with statins, ezetimibe and bempedoic acid achieved LDL-C reductions of approximately 60 % within 6 weeks in a post-ACS patient population [55]. It should be noted, however, that the SANTORINI cohort represents a stable outpatient population, which differs from the ACS settings described above.

The limitations of our study merit further consideration. These include using observational data with limited inference on causality inherent with all such studies, and simulating treatment effects rather than observed changes in a randomised controlled trial. We defined goal attainment as the absolute thresholds of LDL-*C* < 70 mg/dL and <55 mg/dL for high- and very high-risk patients, respectively, but the ≥50 % relative reduction in the 2019 ESC/EAS guidelines was not used due to data constraints, because baseline untreated LDL-C values were not consistently available for the study population [24]. Consequently, we were unable to determine the proportion of patients who would have met this second criterion and the number of patients achieving LDL-C goal in our simulation may have been overestimated.

Although background statin intensity was incorporated into the simulation of LDL-C reductions with bempedoic acid, we did not report simulated CV outcomes stratified by statin intensity. Given that treatment effects may vary according to baseline LDL-C levels and concomitant statin therapy, this may be a potential source of heterogeneity in the estimated effect sizes.

Ezetimibe and bempedoic acid are generally well tolerated; however, treatment discontinuation or suboptimal adherence may occur in routine clinical practice. As this simulation assumed full adherence and persistence with therapy over time, LDL-C reductions and corresponding CV risk reductions estimated in this simulation may be somewhat attenuated in real-world settings.

Furthermore, patients with no documented LLT and patients with missing statin intensity data were excluded, and statin intensification was not simulated because it was assumed that this was stable at the 1-year follow-up. Therefore, we did not conduct a statin optimisation step, as performed by Brandts et al., [18] who did not show significant improvements in goal attainment among patients at high and very high CV risk after statin optimisation. Instead, their simulation suggested that this occurred with the addition of ezetimibe and PCSK9i. We also did not compare the addition of PCSK9i with ezetimibe and bempedoic acid because it was not within the scope of the study objectives, which focused only on oral LLT escalation.

The EU-Wide Cross-Sectional Observational Study of Lipid-Modifying Therapy Use in Secondary and Primary Care (DA VINCI) study suggested that an additional 20 % of patients (80 % overall) would probably achieve their LDL-C goals if a PCSK9i was used in >60 % of patients not at goal, compared with the two-thirds predicted here used in 50 % of patients not at goal [14]. However, the cost of oral bempedoic acid and ezetimibe vs PCSK9i is likely not equivalent. In very high-risk patients, 60 % of patients would require a PCSK9i on top of statins and ezetimibe to attain LDL-C goals in approximately 80 % of the population. By contrast, the addition of bempedoic acid after statins and ezetimibe in 50 % of patients not at goal in our study would lead to goal attainment in approximately 63 % of patients. Therefore, our study suggests that if ezetimibe and bempedoic acid were used, the addition of PCSK9i would target approximately 37 % of patients not at goal and most likely to benefit.

Among patients with ASCVD, we translated the REACH equation from the available SANTORINI data [33]. To determine the number of vascular beds involved (which is required to calculate risk using the REACH equation), conditions affecting the three vascular beds of coronary arteries, carotid arteries and peripheral arteries were used, based on the available disease history from the SANTORINI study. Theoretical ARR and RRR were only calculated in patients who underwent simulation of optimised therapy; the same simulation in a higher- or lower-risk group may result in different predicted absolute benefits. Therefore, the CV outcomes should be interpreted with caution. Importantly, the REACH score was not used to guide treatment decisions or stratify individual patients, as would typically occur in clinical practice. In combination with the acceptable validation results observed in the SANTORINI dataset (Supplementary Table 4; Supplementary Fig. 4), this supports the use of the REACH score in this context. The application of more recent models (SMART or SMART-REACH) [56,57] was limited by data availability in SANTORINI (e.g., missing values for key predictors). Moreover, the REACH score was previously used in a similar model in the DA VINCI simulation study [14], further supporting the appropriateness of our approach. Finally, we assessed the benefit of add-on oral LLT among patients not at the 2019 ESC/EAS guideline-recommended goals [24], whereas the data suggest that LDL-C lowering is beneficial across all levels of risk in relative terms, and at all levels of LDL-C concentrations [3,38].

## 5 Conclusions

Among patients not at LDL-C goal in current routine practice in SANTORINI, further treatment optimisation with adjunctive use of combination oral therapies (i.e., ezetimibe and bempedoic acid) was predicted to increase the proportion of patients achieving LDL-C goals by more than two-fold, from 28.4 % to 63.1 %. These predicted improvements in LDL-C levels would be expected to translate into lower CV risk, based on modelled established relationships between LDL-C change and risk. Optimising LDL-C control by using oral LLT may enable most patients to achieve their LDL-C goal while identifying a small subset of patients who may require additional lipid-lowering strategies, such as PCSK9is.

## Data availability

De-identified individual patient data and applicable supporting clinical study documents are available on request, depending on circumstances, at https://vivli.org. In cases in which clinical study data and supporting documents are provided according to the sponsor’s policies and procedures, the sponsor will continue to protect the privacy of the clinical study participants. Details on data sharing criteria and the procedure for requesting access can be found at https://vivli.org/ourmember/daiichi-sankyo/.

## Ethical approval

As this was a retrospective analysis of de‑identified patient data, neither informed consent nor ethics approval was required.

## Funding

This study was funded by Daiichi Sankyo Europe GmbH, Munich, Germany.

## CRediT authorship contribution statement

**Kausik K. Ray:** Writing – review & editing, Validation, Supervision, Methodology, Investigation, Conceptualization. **Carlos Aguiar:** Writing – review & editing, Validation, Investigation. **Marcello Arca:** Writing – review & editing, Validation, Investigation. **Derek L. Connolly:** Writing – review & editing, Validation, Investigation. **Mats Eriksson:** Writing – review & editing, Validation, Investigation. **Jean Ferrières:** Writing – review & editing, Validation, Investigation. **Ulrich Laufs:** Writing – review & editing, Validation, Investigation. **Jose M. Mostaza:** Writing – review & editing, Validation, Investigation. **David Nanchen:** Writing – review & editing, Validation, Investigation. **Charles Boachie:** Writing – review & editing, Visualization, Validation, Formal analysis, Data curation. **Ben Lee:** Visualization, Writing – review & editing, Validation, Formal analysis, Data curation. **Jarkko Soronen:** Writing – review & editing, Validation, Conceptualization. **Christian Becker:** Supervision, Writing – review & editing, Validation, Project administration, Methodology, Conceptualization. **Ernst Rietzschel:** Writing – review & editing, Validation, Investigation. **Timo Strandberg:** Writing – review & editing, Validation, Investigation. **Hermann Toplak:** Writing – review & editing, Validation, Investigation. **Frank L.J. Visseren:** Writing – review & editing, Validation, Investigation, Conceptualization. **Alberico L. Catapano:** Writing – review & editing, Validation, Investigation.

## Declaration of competing interest

The authors declare the following financial interests/personal relationships which may be considered as potential competing interests: KKR has received lecture fees from Aegerion Pharmaceuticals, Kowa, Cipla, Algorithm and Zuelling Pharma; grant support, paid to his institution, lecture fees and advisory board fees from Amgen, Regeneron Pharmaceuticals/Sanofi, Daiichi Sankyo and Pfizer; lecture fees and fees for serving on steering committees for trials from AstraZeneca and Eli Lilly; fees for serving on steering committees for trials from Cerenis Therapeutics, the Medicines Company and Esperion; advisory board fees from New Amsterdam Pharma, Akcea Therapeutics, Novartis, Silence Therapeutics, Bayer and Daiichi Sankyo; lecture fees and advisory board fees from Takeda, Boehringer Ingelheim and Dr. Reddy’s Laboratories; consulting fees from Silence Therapeutics and Bayer; grant support and advisory board fees from Merck Sharp & Dohme; fees for serving on a clinical events adjudication committee from AbbVie; and fees for serving as principal investigator for a trial from Resverlogix. CA has received study support from Daiichi Sankyo; honoraria for lectures, presentations, speakers’ bureaus, manuscript writing or educational events from Abbott, Amgen, Daiichi Sankyo, Ferrer, Novartis, Servier and Tecnimede; consulting fees from Abbot, Amgen, Daiichi Sankyo, GSK, Novartis, Seriver and Tecnimede; and support for attending meetings and/or travel from Daiichi Sankyo and Tecnimede. MA has received honoraria, lecture fees or research grants from Aegerion, Akcea, Amarin, Amgen, Amryt Pharma, Chiesi, Daiichi Sankyo, Eli Lilly, Ionis Pharmaceutical, Novartis, Pfizer, Regeneron, Sanofi, Ultragenyx and Viatris. DLC has received study support from Daiichi Sankyo and has received honoraria for lecture fees from Daiichi Sankyo. ME has received study support from Daiichi Sankyo; consulting fees from Lipigon, Organon and Sanofi; and received honoraria for lectures, presentations, speakers’ bureaus, manuscript writing or educational events from Sanofi. JF has received study support from Daiichi Sankyo; honoraria for lectures, presentations, speakers’ bureaus, manuscript writing or educational events from Daiichi Sankyo, Novartis, Sanofi and Servier. UL has received study support from Daiichi Sankyo; consulting fees and honoraria for lectures, presentations, speakers’ bureaus, manuscript writing or educational events from Amgen, Daiichi Sankyo, Novartis and Sanofi. JMM has received study support from Daiichi Sankyo. DN is or has been an investigator for clinical studies sponsored by Amgen, Pfizer, Daiichi Sankyo and Novartis. He has not received any personal fees in cash or in any form from these health industries. CBo, BL, JS and CBe are employees of Daiichi Sankyo. ER has received honoraria for lectures from Amgen, Daiichi Sankyo, Servier and Novartis; payments for attending meetings from Sanofi; and for attending advisory boards from Amarin, Amgen and Novartis; all paid directly to Ghent University. He is also President of the Belgian Atherosclerosis Society, a voluntary role. TS has received study support from Daiichi Sankyo; consulting fees from Nutricia, Orion Pharma and Valio; and received honoraria for lectures, presentations, speakers’ bureaus, manuscript writing or educational events from Amarin, Amgen, Daiichi Sankyo, Duodecim, Finnish Medical Journal, GSK, MSD and Novartis. HT has received study support from Daiichi Sankyo and also served on the steering committee of the SANTORINI study. He also received fees as an advisor and speaker for Daiichi Sankyo. FLJV has received study support from Daiichi Sankyo. ALC, in the last three years, has received honoraria, lecture fees or research grants from Aegerion, Amarin, Amgen, Amryt Pharma, AstraZeneca, Daiichi Sankyo, Esperion, Eli Lilly, Ionis Pharmaceutical, Medscape Education, Menarini, MSD, New Amsterdam Pharma, Novartis, Novo Nordisk, PeerVoice, Pfizer, Recordati, Regeneron, Sanofi, The Corpus, Ultragenyx and Viatris.

## Appendix Supplementary materials

Image, application 1
