# Supplementary material for: Potential impact on cholesterol goal achievement and predicted cardiovascular risk by the addition of ezetimibe and bempedoic acid on top of statins: a simulation from the SANTORINI study
Source: Am J Prev Cardiol. 2026 Mar 24;27:101577. doi: 10.1016/j.ajpc.2026.101577 (PMC13261243; doi:10.1016/j.ajpc.2026.101577)
Supplement: Supplementary file 1 [file mmc1.docx]

**Supplementary material**

**Potential impact on cholesterol goal achievement and predicted cardiovascular risk by the addition of ezetimibe and bempedoic acid on top of statins: A simulation from the SANTORINI study**

**Table of Contents**

[Supplementary Table 1. Risk result in treatment optimisation set (*N*=4467) at the 1-year follow-up. 2](#_Toc224753564)

[Supplementary Table 2. Measures of predictive accuracy for REACH score in SANTORINI (ASCVD patients, overall study population). 3](#_Toc224753565)

[Supplementary Table 3. Patient characteristics of the treatment optimisation set (*N*=4467) at the 1-year follow-up. 4](#_Toc224753566)

[Supplementary Table 4. Lipid-lowering therapy of the treatment optimisation set (*N*=4467) at the 1-year follow-up. 5](#_Toc224753567)

[Supplementary Fig. 1. CONSORT diagram for risk calculation. 6](#_Toc224753568)

[Supplementary Fig. 2. Calibration plot for REACH score in SANTORINI (patients with ASCVD, overall population). 7](#_Toc224753569)

[Supplementary Fig. 3. Goal attainment by CV risk before simulation. 8](#_Toc224753570)

[Supplementary Fig. 4. Treatment optimisation and CV risk simulation study design. 9](#_Toc224753571)

[Supplementary Fig. 5. LDL-C values before simulation in the CV risk simulation population (*N*=4327). 10](#_Toc224753572)

# Supplementary Table 1. Risk result in treatment optimisation set (*N*=4467) at the 1-year follow-up.

| **Measures** | **Approaches used to derive 10-year risk  pre-simulation** | |
| --- | --- | --- |
|  | **REACH, SCORE2, and  SCORE2-OP** | **REACH and SCORE** |
| Patients with non-missing CV risk, *n* | 4327 | 4278 |
| Mean RRR: median (95 % quantiles)  – after ezetimibe simulation | 7.6 % (7.0 %, 8.1 %) | 7.6 % (7.1 %, 8.2 %) |
| Mean RRR: median (95 % quantiles)  – after ezetimibe and BA simulation | 13.4 % (12.4 %, 14.3 %) | 13.4 % (12.4 %, 14.3 %) |
| Mean ARR: median (95 % quantiles)  – after ezetimibe simulation | 2.0 % (1.8 %, 2.1 %) | 2.1 % (1.9 %, 2.3 %) |
| Mean ARR: median (95 % quantiles)  – after ezetimibe and BA simulation | 3.4 % (3.2 %, 3.7 %) | 3.7 % (3.4 %, 3.9 %) |

Abbreviations: ARR, absolute risk reduction; BA, bempedoic acid; REACH, REduction of Atherothrombosis for Continued Health, RRR, relative risk reduction; SCORE, Systematic COronary Risk Evaluation; SCORE2-OP, SCORE2-Older Persons.

# Supplementary Table 2. Measures of predictive accuracy for REACH score in SANTORINI (ASCVD patients, overall study population).

| **Analysis population** | ***N*  Patients** | ***N* Events** | **Measures** | **95 % CI (bootstrap, 1000 replicates)** |
| --- | --- | --- | --- | --- |
| AUC | 7145 | 107 | 0.59 | (0.53, 0.64) |
| Calibration slope β |  |  | 1 |  |
| Calibration-in-the-large α |  |  | <0.0001 |  |
| Sensitivity (optimal threshold)^a^ |  |  | 0.65 |  |
| Specificity (optimal threshold)^a^ |  |  | 0.50 |  |

^a^Predicted risk = 0.014.
Abbreviations: ASCVD, atherosclerotic cardiovascular disease; AUC, area under curve; CI, confidence interval; REACH, REduction of Atherothrombosis for Continued Health.

# Supplementary Table 3. Patient characteristics of the treatment optimisation set (*N*=4467) at the 1-year follow-up.

| **Characteristics** | **Treatment optimisation set (*N*=4467)^a^** |
| --- | --- |
| Age, years, mean (SD) | 65.9 (10.8) |
| Female, *n* (%) | 1246 (27.9) |
| Diabetes mellitus, *n* (%) | 1679 (37.6) |
| BMI, kg/m^2^, mean (SD) | 28.3 (4.9) |
| LDL-C, mg/dL, mean (SD) | 86.1 (29.8) |
| High CV risk, *n* (%) | 1219 (27.3) |
| Very high CV risk, *n* (%) | 3189 (71.4) |
| Primary prevention, *n* (%) | 1083 (24.2) |
| Secondary prevention, *n* (%) | 3384 (75.8) |
| MI, *n* (%) | 1914 (42.8) |
| Unstable angina, *n* (%) | 505 (11.3) |
| Stroke, *n* (%) | 316 (7.1) |
| TIA, *n* (%) | 184 (4.1) |
| FH, *n* (%) | 371 (8.3) |

^a^Patients are also the basis of calculation for the CV risk simulation (excluding 140 patients with missing observed risk).
Abbreviations: BMI, body mass index; CV, cardiovascular; LDL-C, low-density lipoprotein cholesterol;
FH, familial hypercholesterolaemia; MI, myocardial infarction; SD, standard deviation; TIA, transient ischaemic attack.

# Supplementary Table 4. Lipid-lowering therapy of the treatment optimisation set (*N*=4467) at the 1-year follow-up.

| **Treatment** | **Treatment optimisation set (*N*=4467)** |
| --- | --- |
| No statin users at follow-up | 210 (4.7) |
| Statins (any) | 4257 (95.3) |
| Low intensity | 112 (2.6) |
| Moderate/high intensity | 4145 (97.4) |
| Ezetimibe use | 1536 (34.4) |
| Bempedoic acid use | 0 (0) |
| PCSK9i alone | 0 (0) |
| PCSK9i combination | 0 (0) |

Data are presented as *n* (%).

Abbreviation: PCSK9i, proprotein convertase subtilisin/kexin type 9 inhibitor.

**
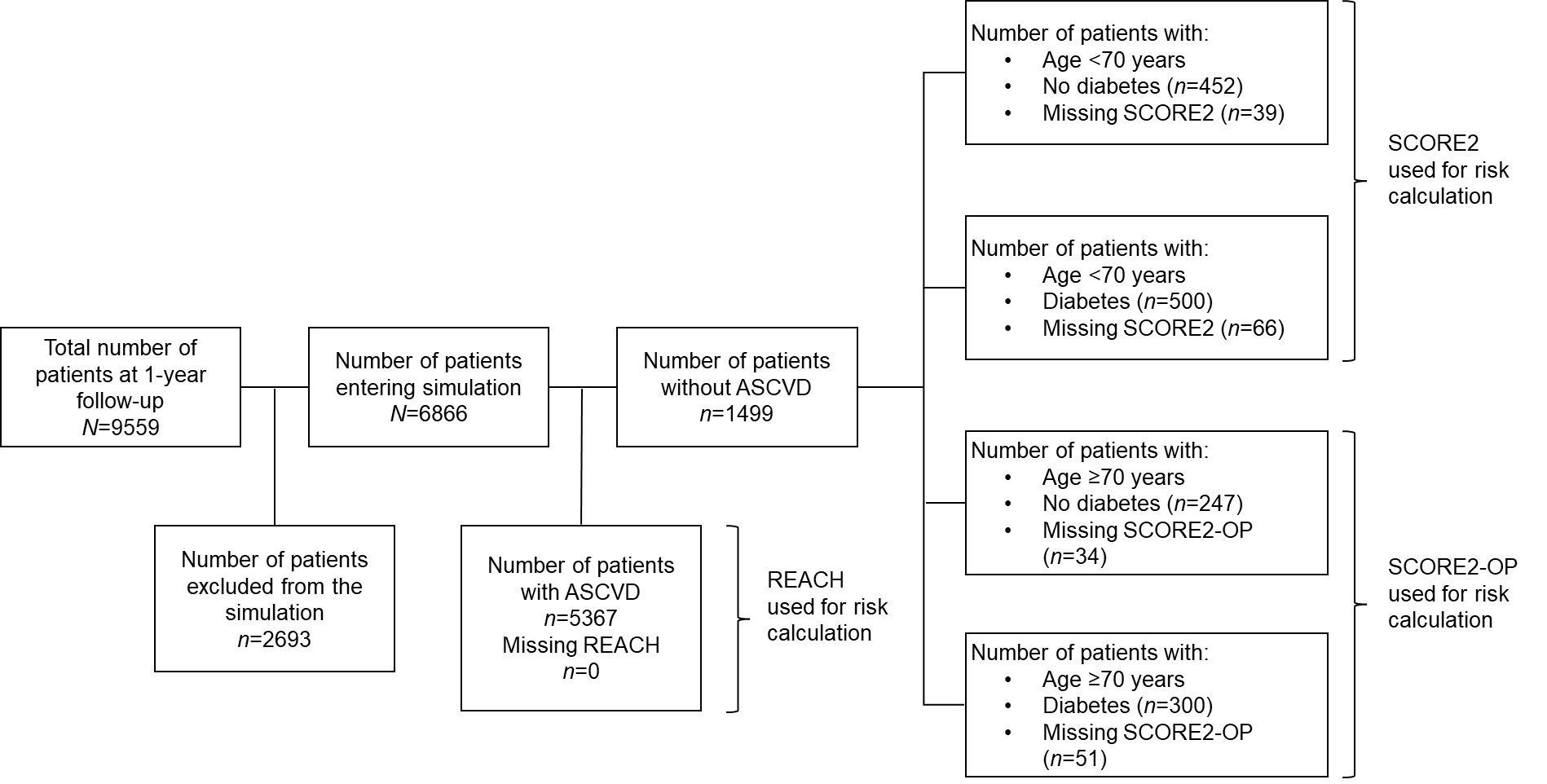
**

# Supplementary Fig. 1. CONSORT diagram for risk calculation.

Abbreviations: ASCVD, atherosclerotic cardiovascular disease; REACH, REduction of Atherothrombosis for Continued Health; SCORE2, Systematic COronary Risk Evaluation; SCORE2-OP, SCORE2-Older Persons.


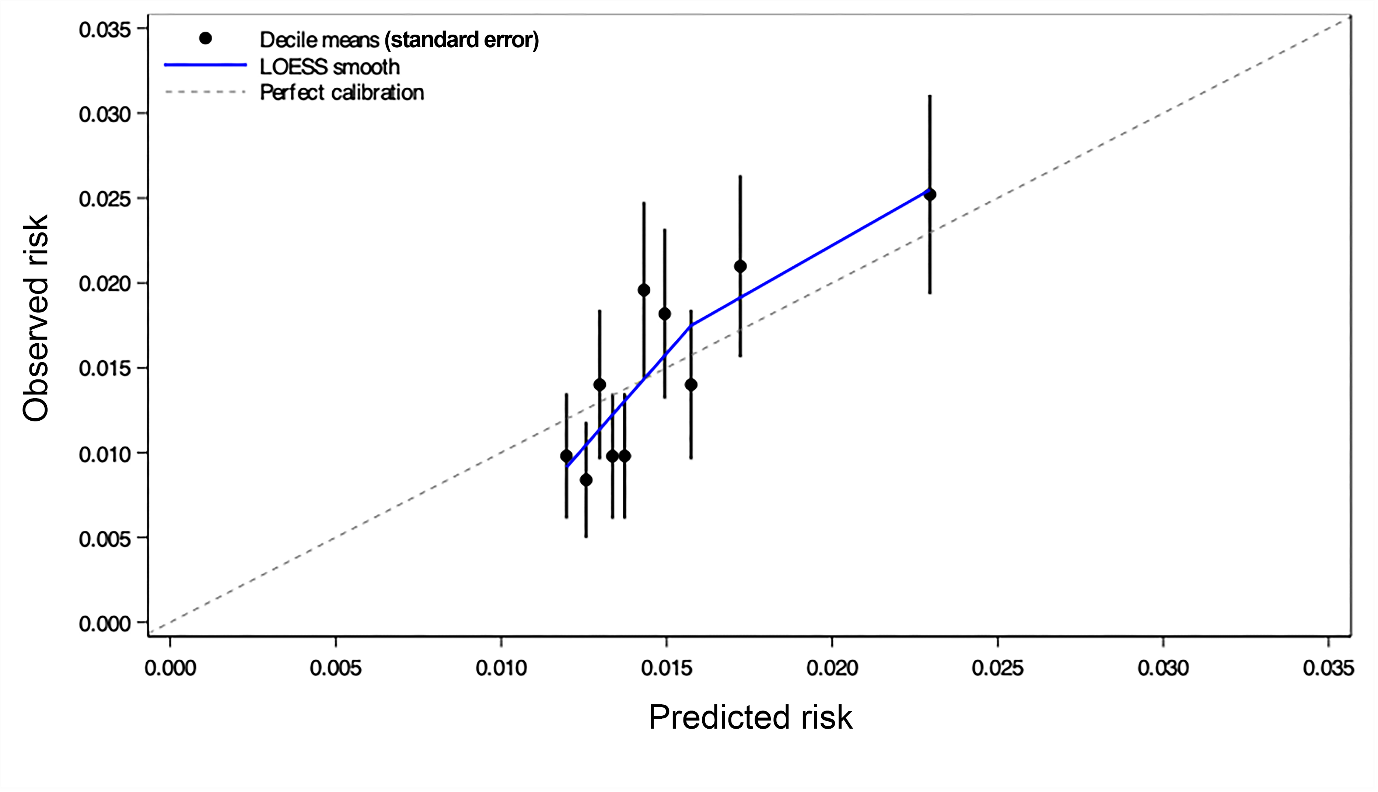


# Supplementary Fig. 2. Calibration plot for REACH score in SANTORINI (patients with ASCVD, overall population).

Abbreviations: ASCVD, atherosclerotic cardiovascular disease; LOESS, locally estimated scatterplot smoothing; REACH, REduction of Atherothrombosis for Continued Health.

**
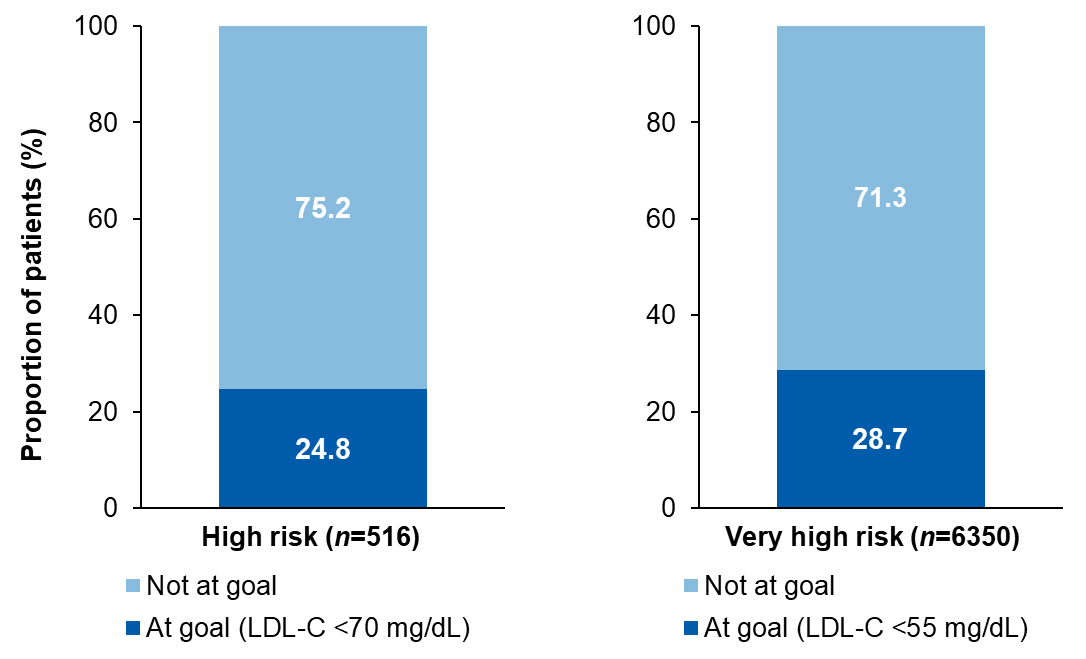
**

# Supplementary Fig. 3. Goal attainment by CV risk before simulation.

Abbreviations: CV, cardiovascular; LDL-C, low-density lipoprotein cholesterol.

**
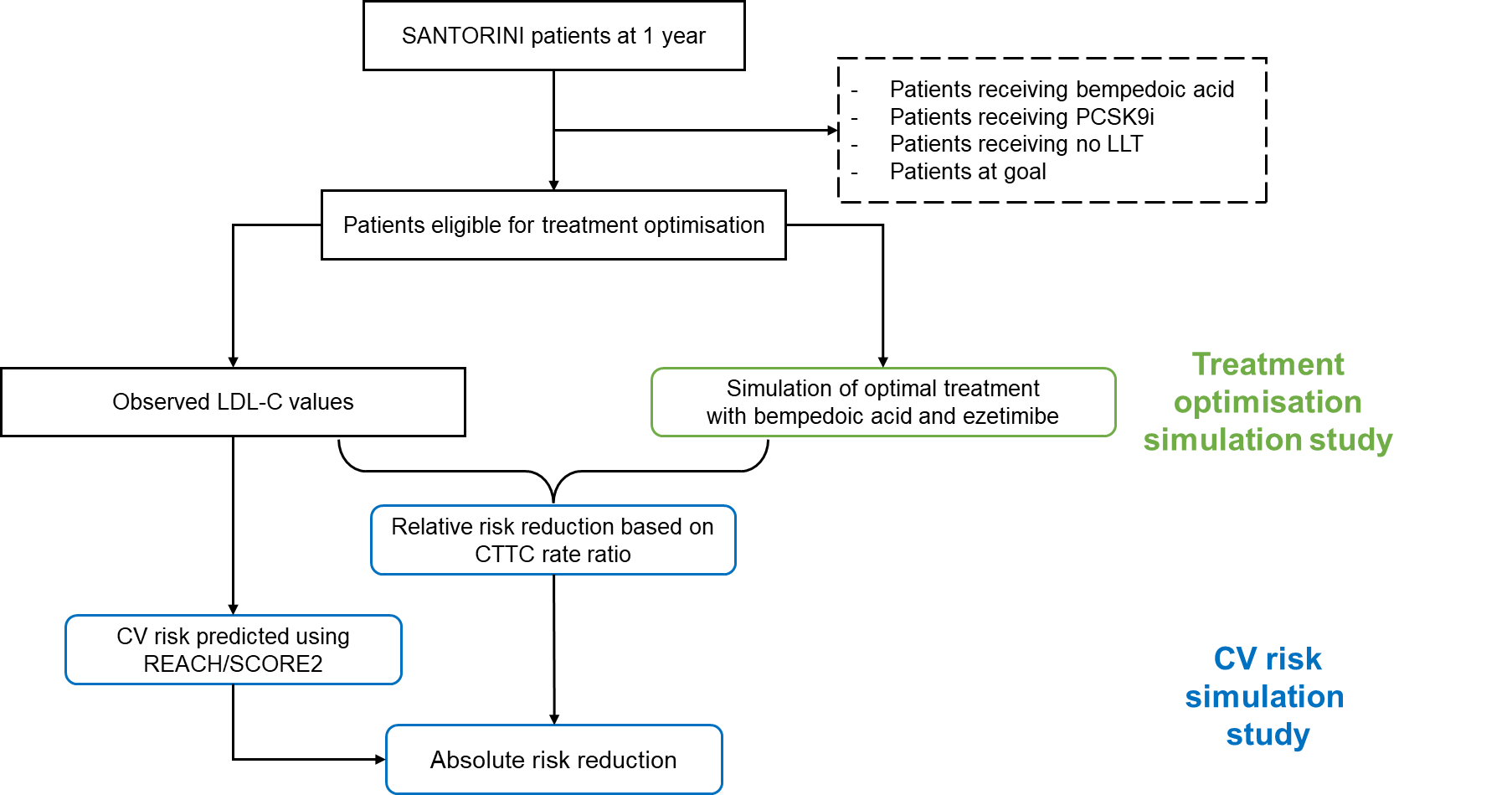
**

# Supplementary Fig. 4. Treatment optimisation and CV risk simulation study design.

Abbreviations: CTTC, Cholesterol Treatment Trialists’ Collaboration; CV, cardiovascular; LDL-C, low-density lipoprotein cholesterol; LLT, lipid-lowering therapy; PCSK9i, proprotein convertase subtilisin/kexin type 9 inhibitor; REACH, REduction of Atherothrombosis for Continued Health; SCORE2, Systematic COronary Risk Evaluation 2.


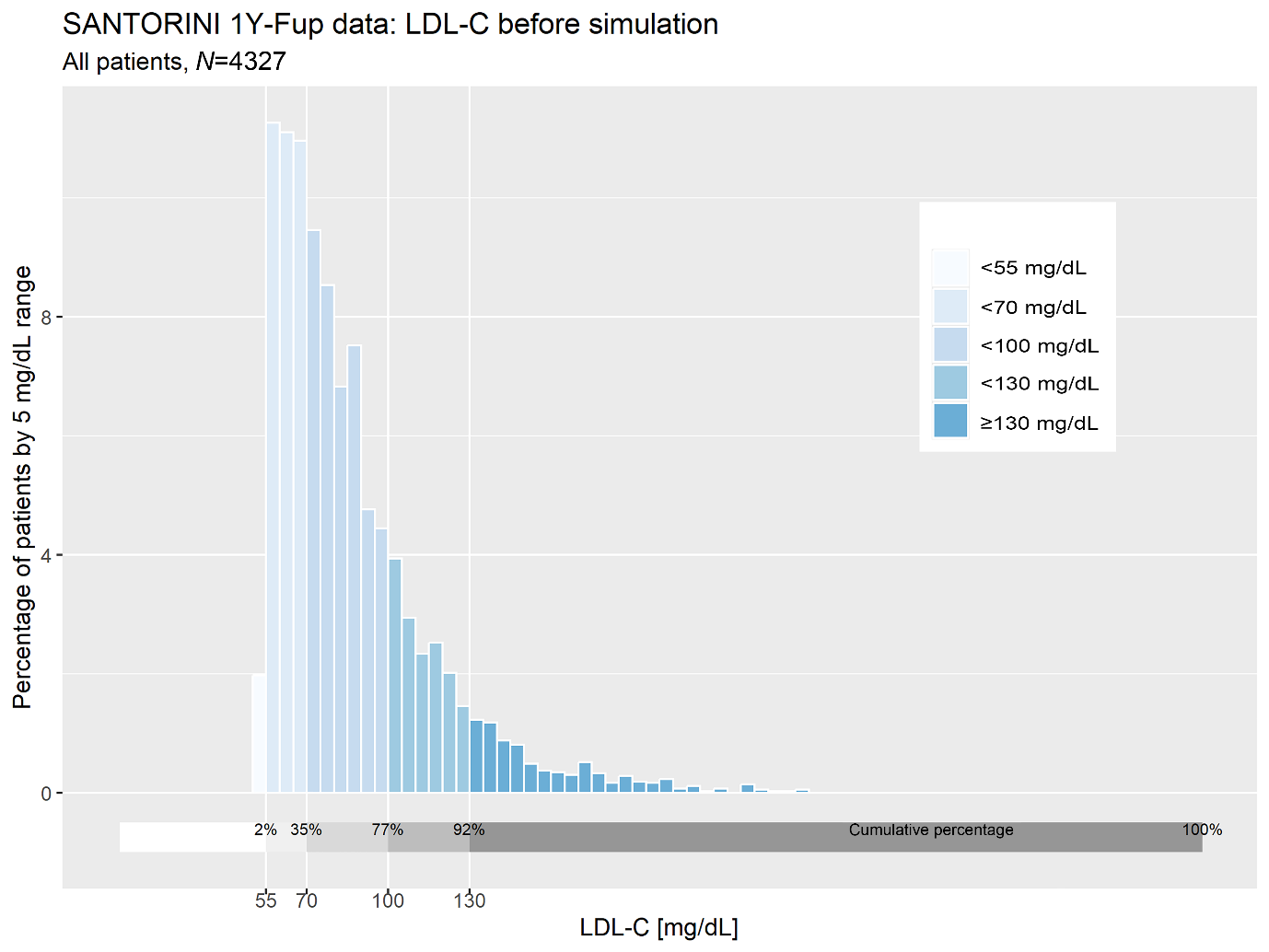


# **Supplementary Fig. 5.** LDL-C values before simulation in the CV risk simulation population (*N*=4327).

Abbreviations: 1Y-Fup, 1-year follow-up; CV, cardiovascular; LDL-C, low-density lipoprotein cholesterol.
